# Systematic Bias in Genomic Classification Due to Contaminating Non-neoplastic Tissue in Breast Tumor Samples

**DOI:** 10.1186/1755-8794-4-54

**Published:** 2011-06-30

**Authors:** Fathi Elloumi, Zhiyuan Hu, Yan Li, Joel S Parker, Margaret L Gulley, Keith D Amos, Melissa A Troester

**Affiliations:** 1 Lineberger Comprehensive Cancer Center, University of North Carolina at Chapel Hill, Chapel Hill, NC 27599, USA; 2 Curriculum in Molecular Biology and Genetics, University of North Carolina at Chapel Hill, Chapel Hill, NC 27599, USA; 3 Department of Pathology and Laboratory Medicine, University of North Carolina at Chapel Hill, Chapel Hill, NC 27599, USA; 4 Department of Surgery, University of North Carolina at Chapel Hill, Chapel Hill, NC 27599, USA; 5 Department of Epidemiology, Gillings School of Global Public Health, University of North Carolina at Chapel Hill, Chapel Hill, NC 27599, USA

**Keywords:** biomarker validation, genomic assays, breast cancer, normal tissue, bias

## Abstract

**Background:**

Genomic tests are available to predict breast cancer recurrence and to guide clinical decision making. These predictors provide recurrence risk scores along with a measure of uncertainty, usually a confidence interval. The confidence interval conveys random error and not systematic bias. Standard tumor sampling methods make this problematic, as it is common to have a substantial proportion (typically 30-50%) of a tumor sample comprised of histologically benign tissue. This "normal" tissue could represent a source of non-random error or systematic bias in genomic classification.

**Methods:**

To assess the performance characteristics of genomic classification to systematic error from normal contamination, we collected 55 tumor samples and paired tumor-adjacent normal tissue. Using genomic signatures from the tumor and paired normal, we evaluated how increasing normal contamination altered recurrence risk scores for various genomic predictors.

**Results:**

Simulations of normal tissue contamination caused misclassification of tumors in all predictors evaluated, but different breast cancer predictors showed different types of vulnerability to normal tissue bias. While two predictors had unpredictable direction of bias (either higher or lower risk of relapse resulted from normal contamination), one signature showed predictable direction of normal tissue effects. Due to this predictable direction of effect, this signature (the PAM50) was adjusted for normal tissue contamination and these corrections improved sensitivity and negative predictive value. For all three assays quality control standards and/or appropriate bias adjustment strategies can be used to improve assay reliability.

**Conclusions:**

Normal tissue sampled concurrently with tumor is an important source of bias in breast genomic predictors. All genomic predictors show some sensitivity to normal tissue contamination and ideal strategies for mitigating this bias vary depending upon the particular genes and computational methods used in the predictor.

## Background

Breast cancer is well-recognized as a heterogeneous disease and great progress has been made in the past decade in classifying tumors for prognosis and prediction [1-8]. Two different assays are clinically and commercially available for genomic characterization of tumors: the 21-gene OncotypeDx assay (Genome Health Inc, Redwood City, CA) for estrogen receptor (ER)-positive, early stage breast cancer [6,7] and the 70-gene Mammaprint (Agendia, Huntington Beach, CA) assay [4,5] for ER-positive and ER-negative early-stage, node-negative breast cancers. A 50-gene subtype predictor, the PAM50 [8], has been validated for stratifying node-negative patients according to prognosis and tumor subtype. Each of these three assays results in a clinically useful score, with OncotypeDx providing a continuous but categorizable recurrence score, Mammaprint providing a dichotomous high risk or low risk categorization, and PAM50 providing a continuous and categorizable risk of relapse (ROR) score, along with a categorical classification of biological subtype. These scores are computed based on the expression of selected transcripts in a heterogeneous tissue sample comprised of varying amounts of malignant cells, tumor stroma, and histologically normal breast tissue. The tumor-adjacent normal breast tissue contributes RNA that dilutes the malignant cell RNA in a sample. Because normal tissue and tumor tissue have markedly different expression patterns [9], this could be an important source of non-random error in genomic predictors.

Random error is error due to chance, can be in either direction, and has been operationally defined as "unexplained variation". This type of error can cause estimates that vary in either direction and have an average or net effect of zero [10]. Random error results in imprecision, but precision can be improved by replication. Non-random or systematic error is a bias that has a net direction and magnitude, and importantly, this bias does not diminish with replication. Systematic error or bias can have magnitudes that are as large as or larger than the effect of interest. In the context of genomic predictors, normal tissue could have effects on gene expression that are similar in magnitude to differences between subtypes. The bias could also be in a consistent direction (for example biasing the predictor toward a lower recurrence risk) or could be unpredictable with no consistent direction. The magnitude and direction of normal tissue effects on existing genomic predictors have not been evaluated.

To investigate how normal tissue contamination of tumor samples affects genomic predictions, we analyzed 55 samples of breast cancer and paired adjacent normal tissue from the same patients [9]. By combining signal from normal and tumor specimens, we performed a sensitivity analysis, evaluating how normal contamination affected tumor classifiers. Our results show that depending upon the specific predictor, the direction of bias was either predictable (tending in the same direction) or unpredictable. Predictable bias allows for correction, and we identified low variability across normal tissues and high variability across tumors as a desirable feature of genomic predictors with predictable bias. We then conducted a sensitivity analysis of the vulnerability of genomic assays to contamination bias by applying median normal tissue gene expression levels in a linear model to adjust for contamination bias, demonstrating that adjustment for contamination improves sensitivity and negative predictive value.

## Results and Discussion

### Effects of normal contamination in tumors with paired normal tissue

**Figure 1:**
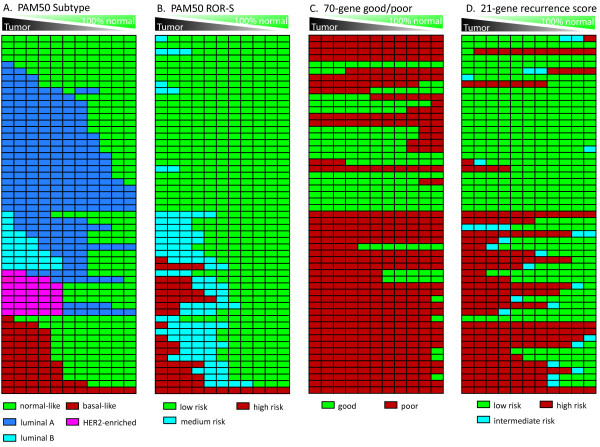
**Tumor classification for the 55 paired normal and tumor samples using (A) PAM50 intrinsic subtype predictor, (B) PAM50 Risk of Recurrence (ROR-S), (C) 70-gene good/poor prognosis signature (Mammaprint), and (D) 21-gene recurrence score predictor (Oncotype DX)**. The leftmost column in each panel shows the unadjusted classification, while subsequent columns show the effects of increasing normal tissue sampled with the tumor. Pure normal tissue gene expression is shown in the right column of each panel. Color coding is as follows: (A) Normal-like, green; Luminal A, dark blue; Luminal B, light blue; HER2E, magenta; and Basal-like, red (B) PAM50 ROR-S score low-risk, green; medium-risk, light blue, and high-risk, red (C) Prognosis score: good, green; poor, red. (D) 21-gene assay: low, green; intermediate, light blue; and high, red.

Normal tissue in tumor specimens altered the prediction of tumor subtype or prognosis. Figure 1 shows that in 55 pairs of tumor and normal tissue, the genomic prediction changed as normal tissue contribution to gene expression increased. In the case of the PAM50 subtype predictor (Figure 1A), the samples moved from more aggressive subtypes to less aggressive subtypes as the normal contents increased. The same is true of the ROR-S (Figure 1B). The bias induced by normal contents increased the likelihood of underestimating patient risk. However, the PAM50 was the only assay that showed predictable direction of misclassification.

The other two predictors did not show predictable changes in risk score with increasing percentage normal. With the 70-gene good/poor prognosis and 21-gene recurrence score assays (Figure 1C and 1D respectively), normal contamination biased subtype classification in either direction. For example, 9 samples that were classified as good prognosis using the tumor samples were classified as poor prognosis as normal percentage increased, while for 11 samples increasing normal changed the prediction from poor to good (Figure 1C). The 21-gene recurrence score also caused some samples to move from high to intermediate to low risk, while others moved from low to intermediate to high risk as normal percentage increased (Figure 1D). There was also substantial variation in the genomic classification of the 100% histologically normal tissue. In the final column of Figure 1C and 1D, normal tissue in the 70-gene signature is called poor prognosis in 67 percent of cases and normal tissue in 21-gene signature is high or medium risk in 17 percent of cases.

**Figure 2:**
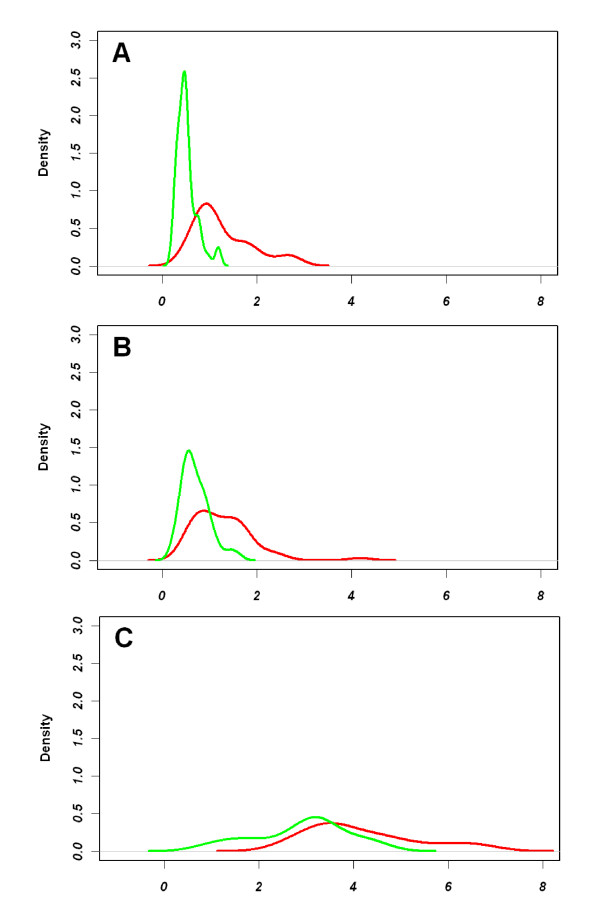
**Interquartile range distribution for normal tissue and paired tumor tissue across three predictors: (A) PAM50, (B) 70-gene good/poor prognosis, and (C) 16 non-housekeeping genes in the 21-gene recurrence score (C)**. The interquartile range distribution is green for cancer-adjacent normal and red for tumors.

The differences in predictability of bias direction and the prevalence of high risk phenotype in normal tissue between the PAM50 and the other predictors can be explained by qualitative differences in variability of each gene set across normal samples. Due to the bioinformatics methods used to develop the PAM50 (i.e. intrinsic subtype algorithms[2]), the variation and interquartile range for the PAM50 genes is very low in normal tissues, while it is high across different tumor specimens (Figure 2A & Additional File 1, Figure S1). High variation in tumor samples allows good dynamic range in distinguishing the different tumor specimens from one another, while the low variation in the normal samples means that normal tissue contamination tends to lead to similar effects across samples. Normal tissue appears to have greater variability in the 70-gene (Figure 2B) and 21-gene assays (Figure 2C), even showing similar variability to the tumor themselves in the 21-gene assay.

### Effects of Normal Contamination in Public Datasets

Biospecimens of histologically normal tissue adjacent to tumor are rarely available for paired analyses like those performed in the current study, so a generic or prototypical normal profile that characterizes the average effect of normal contamination on tumor predictors could be useful in assessing bias or in adjusting tumor classifications to account for normal contamination. It was possible to define a prototypical normal profile because approximately 90% of samples had normal-like subtype by the PAM50 assay (see Figure 1A) and showed highly correlated gene expression (93% of correlation coefficients greater than 0.6) and low variability across normal samples (Figure 2A). It has previously been reported[11] that a small percentage of normal tissue adjacent to cancer can express a profile similar to invasive ductal carcinomas, and this was also observed in our dataset, but for only a minority (11%) of samples. We excluded these tumor-like normal samples in developing a prototypical normal contamination profile and used 48 normal-like samples to calculate median gene expression. We compared results using this median or protypical cancer-adjacent normal to those obtained using the paired cancer-adjacent normal, and found that subtype classifications under various normal contamination scenarios ranging from 0 to 50% are similar using either the median normal or paired normal tissue (Additional File 2, Figure S2).

### Correcting for normal percentages in application of the PAM50

The tumor sample profile is a mixture between the malignant cell profile and its adjacent or contaminating normal profile. Using the prototypical normal profile to deconvolute these two profiles for the only assay with predictable direction of bias (PAM50), we applied Equation 2 (see Methods) to correct the subtype of 24 patients where the percentage of tumor cells, *a*, was carefully estimated. Five of the 24 samples had original subtype classifications that differed from those after correction for percentage normal (Additional File 3, Table S1). Applying the prototypical normal to these specimens with known percentage tumor did result in changes to subtype with one normal-like sample (with 90% normal contamination) and 3 Luminal A samples (with 70%, 30%, and 25% normal) being reclassified as Luminal B after correction. One Luminal B sample (50% normal) was reclassified as a Her2-enriched (HER2E) subtype. Looking at the ROR-S score, 8 of 24 patients changed in score, with 4 patients switching from Low to Medium or High risk and 4 patients switching from Medium to High risk after adjustment. No patients moved to a less aggressive ROR-S score.

We next turned to three large public datasets to evaluate how corrections based on the PAM50 prototypical normal profile would influence subtype, ROR-S, sensitivity and negative predictive value. In these larger public datasets, the true percentage of normal is not known for individual samples, but tumors were sampled with quality control criteria that ensured > 50% tumor cellularity[5], > 70% tumor cellularity[12], or a median percent tumor cellularity of 60% was reported[13]. We applied different correction rates from 5 to 30%, 40%, or 50% (by 5% increment with maximum correction dictated by the published quality controls in place for each dataset) across node negative samples and estimated the PAM50 subtype and ROR-S at each normal correction rate. Table 1 shows that the distribution of subtype and ROR-S score shifts toward more aggressive subtypes after adjusting for contaminating normal. In NKI, normal-like and Luminal A subtypes moved to more aggressive Luminal B or HER2E tumors and low and medium risk samples moved to a higher risk class after adjusting for normal bias. The Luminal B, HER2E, Basal-like subtypes and ROR-S high risk classes were most tolerant to normal contamination. The Naderi et al.[13] and Wang et al.[12] datasets also showed similar trends toward more aggressive subtypes after adjusting for normal contamination.

**Table 1:** Adjusted and unadjusted subtype, ROR-S score (node negative patients)

	Unadjusted, n	Adjusted^a^, n
**NKI**		
Normal-like	5	2
Luminal A	85	61
Luminal B	18	41
HER2-enriched	14	18
Basal-like	29	29
		
Low	78	48
Medium	43	49
High	30	54
		
**Naderi et al.**		
Normal-like	6	2
Luminal A	43	35
Luminal B	15	24
HER2-enriched	11	12
Basal-like	11	13
		
Low	39	25
Medium	27	33
High	20	28
		
**Wang et al.**		
Normal-like	15	3
Luminal A	138	58
Luminal B	51	128
HER2-enriched	29	40
Basal-like	53	57
		
Low	128	39
Medium	85	122
High	73	125

^a^NKI was adjusted for 30% normal, Naderi et al. was adjusted for 20% normal, and Wang et al. was adjusted for 10% normal.

**Figure 3:**
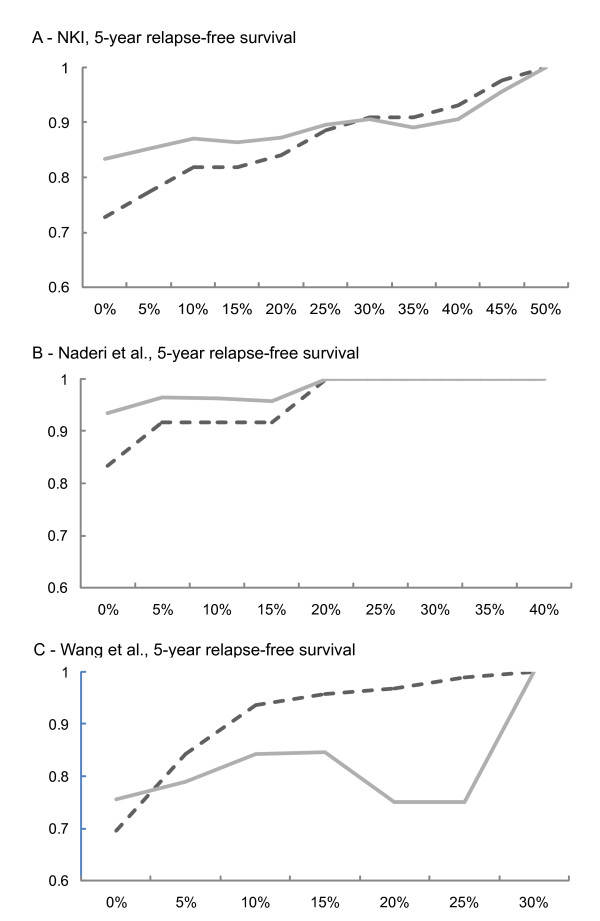
**Sensitivity (dashed line) and negative predictive value (solid line) at five years for (A) NKI (B) Naderi et al., and (C) Wang et al**. The sensitivity and negative predictive value (NPV) are plotted as a function of percentage normal correction (equation 2) with the percentage normal set at 5% increments from 0 to 30-50%. Sensitivity and NPV were defined in the methods section comparing low to medium-high (taken together) ROR-S classes from the PAM50 assay.

If the true subtype is identifiable by adjusting for normal contamination, then the sensitivity and negative predictive value should increase after correction. Figure 3 shows the change in sensitivity and negative predictive value (NPV) as a function of correction rate in the NKI, Naderi et al., and Wang et al. datasets. In all three datasets, the sensitivity in detecting patients with relapse-free survival < 5 years increased after adjustment for normal contamination and the negative predictive value increased. For each dataset, the maximum % correction shown is consistent with the maximum percentage of normal allowed (for NKI and Wang et al.) or the median % normal reported (Naderi et al.). Sensitivity and negative predictive value are also given for 10-year and relapse-free survival at the end of follow up for all three datasets in Additional File 4, Supplementary Data 1 (excel file). In predicting 5-year relapse-free survival, NPV and sensitivity tended to increase with increasing normal adjustment.

Different correction rates were needed to attain maximum NPV with different datasets. This reflects the fact that contamination rates differed for each of these populations; different quality control criteria were stated for each dataset, and the optimum percentage correction correlated with the stated tumor purity. The NKI dataset had the most inclusive tumor quality control criteria (> 50% tumor nuclei) and required the most aggressive corrections (30% adjustment), while the Naderi et al. dataset with median 60% tumor nuclei required 20% correction and the Wang et al. dataset had the most stringent quality controls (> 70% tumor nuclei) and required the least adjustment (10%). To further confirm that samples with lower contamination rates produce more stable estimates, we used a fourth public dataset of microdissected samples. This fourth dataset had too few samples to perform survival analyses and NPV calculations, but using 48 samples with > 90% tumor cellularity[14], we applied 5% and 10% normal correction. As expected, very few samples changed subtypes with normal correction in this dataset. Only one sample (2% of samples) changed subtype at 5% normal correction and four samples (8% of samples) changed at the more aggressive 10% correction rate. Thus, the misclassification evident using normal adjustment was correlated with reported contamination severity for these datasets.

**Figure 4:**
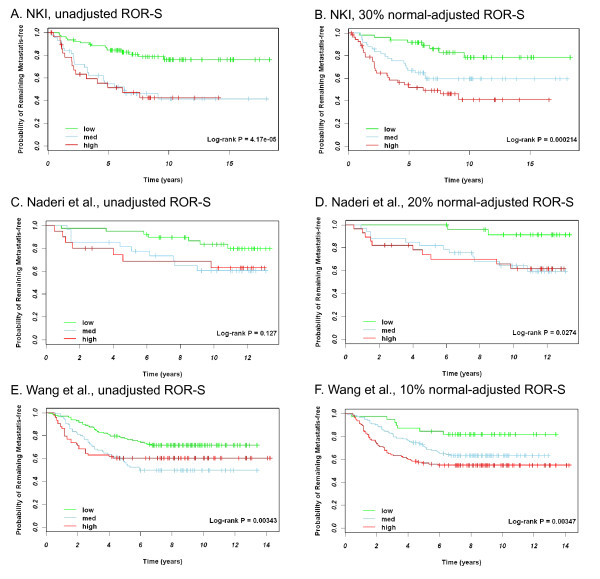
**Relapse-free Survival plots for PAM50 ROR-S score given (A) unadjusted NKI data, (B) 30% normal-adjusted NKI data (C) unadjusted Naderi et al. (D) 20% normal-adjusted Naderi et al. data, (E) unadjusted Wang et al. and (F) 10% normal-adjusted Wang et al. data**. Corrections to the expression assuming a given percentage of tumor (*a*) were calculated using equation 2. In each figure, the green line is low ROR-S, the blue line is medium ROR-S, and the red line is high ROR-S.

Normal-contamination correction improves the relapse-free survival curve separation for node-negative patients (Figure 4). Unadjusted Kaplan-Meier curves are shown along with curves adjusted for normal contamination. Additional File 5, Figure S3 shows overall survival in node-negative patients from NKI and Naderi et al., but overall survival was unavailable for the Wang et al. dataset. Across all of these survival analyses, the qualitative trends are the same: (1) normal contamination in tumor specimens causes more aggressive tumors to be misclassified as less aggressive and (2) adjustment for normal contamination improves negative predictive value.

## Conclusions

This paper highlights complex, but fundamental, issues related to validation of tissue-based genomic biomarkers. In our assessments of the published methods for several clinically-relevant genomic assays, we found that normal contamination is an important source of bias in genomic predictors. However, contaminating normal tissue has different types of impact depending upon the genes included in the assay. While the 70-gene assay provides stable results at high tumor percentage, unpredictable bias occurs when tumor percentage is low (< 70%). The 21-gene assay also showed unpredictable direction of bias due to contaminating normal. Both of these assays, in their commercial forms, have implemented quality control strategies to account for tumor nuclei content. These strategies appear important given that these assays misclassify a large number of non-neoplastic specimens as more aggressive tumor types. For the PAM50 assay, contaminating normal tissue induced predictable and unidirectional changes in subtype. The PAM50 genes have low variability within normal tissue and distinct expression between normal and tumor tissue, perhaps because of the way in which these genes were selected: by identifying genes that had high variation between different tumors and low variation between different samplings of the same specimen[2]. To correct for normal contamination in the PAM50 assay, we calculated the median normal expression across each of the PAM50 genes and applied a simple, linear correction to several datasets representing more than 800 breast cancer patients. Our results demonstrate that computational approaches adjusting for normal tissue contamination bias can improve the predictive value of PAM50 genomic classification.

A few previous studies have attempted to identify gene expression signatures reflective of pure tumor cells [15-23] or associated with percentage of stroma in tumor [23]. Other studies have used microdissection to isolate or enrich for malignant epithelial cells [24]. Our results suggest that identifying genomic predictors that quantitatively estimate percent normal in tumor specimens is a challenging problem; we were unable to validate previous signatures [23] or identify a new signature that accurately predicts normal contamination in independent datasets. Until such a signature is identified and validated, it will remain difficult to implement correction strategies for individual patients. However, to illustrate the importance of the problem we have used public data to conduct a careful sensitivity analysis of the potential for normal contamination to affect genomic assay results. Our sensitivity analysis was designed to evaluate a plausible scenario for the effects of normal contamination, but actual effects of a given percentage of normal may be over-estimated for some samples in these datasets. This over-estimation of the effects could arise from differences in the yield of RNA per cell between normal and malignant cells [25], such that histologically evaluated percentage normal does not correspond linearly with a similar percentage change in the gene expression. In fact, for the Naderi data where median tumor cellularity was 60%, only 20% correction was required, suggesting that normal contamination contributes less RNA per cell, or that the signatures are robust to some percentage contamination, or both. Pathologic evaluation of percentage tumor cellularity is also subject to inter-rater variability. However, by assuming 1:1 yield between normal and tumor, the current study shows a plausible worst-case scenario of the biasing effects of a given percentage of normal tissue and highlights how vulnerability may vary across different genomic assays.

Future work should assess the strengths and weaknesses of various strategies for dealing with normal contamination, ranging from pathologist review and dissection to genomic methods for assessing and correcting for normal bias. Microdissection or other methods for gross dissection may be necessary for some assays with genes that are highly variable in normal tissue or stroma. Alternatively, the development of preanalytic criteria, such as requiring a minimal percentage of malignant cells in a particular sampling may remain important for ensuring quality results. The commercial version of the 70 gene assay implements such preanalytic criteria. One disadvantage of requiring high levels of tumor nuclei is that some samples will be excluded. Patients who are likely to be excluded are those who have small tumors at the time of detection. For some assays, computational adjustment may obviate the need for microdissection. For assays with low variation in normal and predictable direction of effect, pathologist evaluation of percentage tumor remains useful in determining the likelihood of normal contamination bias or in identifying bounds on correction rates, but less labor intensive sampling strategies may be possible to minimize the cost of these assays.

For the PAM50 assay, the tolerance to contamination by normal tissue is greatest for Luminal B, HER2E, and Basal-like subtypes or High risk classes. For these assays, the tumor signature is still strongly evident at even low percentages of tumor. The observation of more stable classification for Basal-like breast cancers coincides with the recent observation that Basal-like breast cancers are more robustly identified (relative to other subtypes) in single sample predictors[26]. Conversely, this also demonstrates that accurate identification of Luminal A tumors is dependent upon having high malignant cell percentages (i.e. low levels of normal contamination). The majority of these erroneously classified Luminal A tumors are Luminal B after adjustment for normal contamination, suggesting that Luminal B tumors may "masquerade" as Luminal A tumors due to the presence of high levels of normal tissue in the specimen. This observation is particularly important because misclassification could lead to undertreatment if this error is not modeled and corrected.

It has been argued that the scientific rigor of translational biomarker research has lagged behind that of treatment research [27] and that second generation genomic tests should deal with limitations of the first generation tests, including the need for higher levels of evidence [28]. Our results suggest that desirable features of second generation tests will include attention to important sources of preanalytic variation in tumor specimens, including normal contamination and its quantitative effects in biasing tumor classification. Discussion of these biases, including direction, magnitude, predictability, and thorough assessment of the assay sensitivity to these biases are important considerations. It is not the case that a given assay is simply resistant or vulnerable to normal contamination, but rather, the particular genes in an assay create complex patterns of bias, that must be further characterized. Other sources of variation in biospecimen processing [29-31] should also be carefully considered using similar sensitivity analyses. The next generation of genomic tests for clinical stratification of breast cancer patients will make important improvements upon the currently available tests by attending to these important variables.

## Methods

### Samples and clinical data

We used new data from tumors, tumor-normal pairs, and public data for this study. The new data on tumor and tumor-adjacent normal tissue pairs were from patients undergoing surgery for invasive breast cancer at UNC Hospitals. We define normal as benign tissue, whether normal-appearing, reactive, or desmoplastic. Gene expression microarrays were performed on both tumor and adjacent normal tissues for 55 tumor-normal pairs from the same patient, and 24 additional tumor samples (referred to as UNC24 in tables and text below) were obtained where tumor cell percentage was quantified by counting malignant and normal nuclei in a hematoxylin and eosin (H&E) stained paraffin sections. The H&E stained frozen section was made from a mirror image tissue specimen immediately adjacent to the frozen tissue used for expression analysis. All of these samples were collected with patient informed consent by the UNC Lineberger Tissue Procurement Facility under an Institutional Review Board approved protocol[9]. Public datasets representing approximately 800 patients and more than 500 node-negative patients were analyzed for this study, including datasets from the Netherlands Cancer Institute (NKI 295)[5], Nottingham City Hospital NHS Trust presented in Naderi et al.[13], Erasmus Medical Center, Rotterdam, Netherlands Wang et al.[12] and University Hospital La Paz, Madrid Spain as described in Natrajan et al.[14]. Clinical characteristics of patients in each dataset are presented in Table 2.

**Table 2:** Patient characteristics according to expression data set

	Tumor and normal pairs	UNC set, known % normal (UNC24)	NKI 295	Naderi et al.	Wang et al.	Natrajan et al.
Patients (n)	55	24	295	135	286	48
Microarrays (n)	118	24	295	307	286	48
Median RFS, months	14	31	78.2	119.5	86	NA
missing RFS, n (%)	33	6	0	1	0	48
						
Estrogen receptor status, n (%)						
positive	35 (63.6)	15 (62.5)	226 (76.6)	93 (68.9)	209 (73.1)	25 (52.1)
negative	18 (32.7)	7 (29.2)	69 (23.4)	40 (29.6)	77 (26.9)	23 (47.9)
missing	2 (3.6)	2 (8.3)	0 (0)	2 (1.5)	0 (0)	0 (0)
						
AGE, years						
mean (standard deviation)	53.2 (13.9)	59.8 (17.0)	44.0 (5.5)	56.9 (8.9)	54 (12)	55 (12.6)
						
GRADE, n (%)						
1	5 (9.1)	2 (8.3)	75 (25.4)	35 (26.0)	7 (2.0)	0 (0)
2	16 (29.1)	7 (29.2)	101 (34.2)	50 (37.0)	42 (15.0)	0 (0)
3	19 (34.5)	14 (58.3)	119 (40.3)	49 (36.3)	148 (52.0)	48 (100.0)
missing	15 (27.3)	1 (4.2)	0 (0)	1 (0.7)	89 (31.0)	0 (0)
						
TUMOR SIZE, n (%)						
< = 2	17 (30.9)	17 (70.8)	155 (52.5)	93 (68.9)	278 (0)	18 (37.5)
> 2	38 (69.1)	6 (25)	140 (47.5)	41 (30.4)	8 (0)	30 (62.5)
missing	0 (0)	1 (4.2)	0 (0)	1 (0.7)	0 (0)	0 (0)
						
NODE, n (%)						
positive	1 (1.8)	14 (58.3)	144 (48.8)	43 (31.9)	0 (0)	23 (48.0)
negative	54 (98.2)	9 (37.5)	151 (51.2)	86 (63.7)	286 (100)	24 (50.0)
missing	0 (0)	1 (4.2)	0 (0)	6 (4.4)	0(0)	1 (2.0)
						
TUMOR SUBTYPES, n (%)						
normal	4 (7.3)	1 (4.2)	8 (2.7)	13 (9.6)	15 (5.2)	1 (2.0)
LumA	23 (41.8)	6 (25.0)	167 (56.6)	59 (43.7)	138 (48.3)	17 (35.4)
LumB	9 (16.4)	9 (37.5)	51 (17.3)	25 (18.5)	51 (17.8)	8 (16.7)
Her2	7 (12.7)	3 (12.5)	23 (7.8)	21 (15.6)	29 (10.1)	9 (18.8)
Basal	12 (21.8)	5 (20.8)	46 (15.6)	17 (12.6)	53 (18.5)	13 (27.1)

### Microarrays

Total RNA was extracted from tumor and paired tumor-adjacent normal tissue by homogenizing tissue and then using an RNeasy RNA extraction kit (Qiagen) to isolate total RNA. RNA quality was assessed using an Agilent bioanalyzer and only samples with RIN > 7 were used for subsequent microarrays. Agilent custom 244 k or catalog 4 × 44 k arrays were performed with linear amplification and labeling according to manufacturer protocol. Two-color Agilent protocols were used for all samples, with Cy3-labeled reference produced from total RNA from Stratagene Universal Human Reference (spiked 1:1000 with MCF-7 RNA and 1:1000 with ME16C RNA to increase expression of breast cancer genes) and Cy-5 labeled patient specimens. New data are publicly available through the Gene Expression Omnibus (GSE22384).

### Tumor-Normal Mixing

The impact of increasing proportions of adjacent normal tissue was assessed for each sample using linear combinations of tumor gene expression and paired normal gene expression according to the following:(1)

where *a *is the percentage of tumor signal and 1-*a *is the percentage of normal signal. Combinations were made with increments of *a *equal to 0.10. The assumption that RNA level has a linear and symmetrical influence on transcript abundance for the PAM50 genes was tested experimentally. Specifically, expression data from pure populations of cancer cells grown in monoculture were linearly combined with expression data from pure populations of breast fibroblasts; expected expression values for ratios of epithelium to stroma equal to 3:1, 2:1 or 1:1 were computed. These computed values were compared to observed gene expression for cocultured cells where the actual percentage of epithelial content was 75%, 66% purity, or 50%, respectively. All data for these analyses are available in the Gene Expression Omnibus, GSE26411) and described in Camp et al. [32]. Additional File 6, Table S2 shows that the correlations between observed and computed were high (0.80-0.92) and that the slopes were very close to 1, suggesting linear and symmetrical influence of epithelium and stroma on transcript abundance.

### Normalization and Classification with Genomic Predictors

Data were Lowess-normalized and genes that had a signal of < 10 dpi in either channel were excluded as missing, and genes that had more than 30% missing data across all samples were excluded from further analysis. For genes that passed this filter, missing data were imputed using 10 nearest neighbors. The following genomic predictors were applied to these data based on published methods: the good/poor prognosis signature of Van't Veer et al.[4,5] that is currently the basis for the Mammaprint assay by Agendia, the recurrence score 21-gene assay that is the basis for Oncotype DX[6,7], and the recurrence risk (ROR-S) and subtype predictions based on the PAM50 assay[8]. The versions of these assays applied in this paper are based on the published scientific literature and may differ from the patented versions of these assays that are in clinical use. However, subtle differences between patented and published versions of the assay are unlikely to substantially reduce the validity of the sensitivity analyses performed in this work.

For the good/poor prognosis predictor we used the set of probes from the original 70 probes that were available in our platform and in the 55 paired sample dataset. The 70-probe signature contains 60 unique genes, and 95% of these (57 of 60) were on our array. Implementation of the 70 gene good/poor prognosis and 21-gene recurrence assays on NKI data resulted in highly significant prognosis predictions (Additional File 7, Figure S4) suggesting that the current dataset represents tumors with similar clinical characteristics as previous tumor datasets. Applying the PAM50 to the 55 tumor samples gave representatives of all five subtypes, while the paired normal tissue was predominantly of normal-like subtype. A small percentage of non-normal-like subtype specimens was expected based on previous studies that have shown up to 10% of histologically normal specimens from cancer patients may have invasive-like signatures [11]. Of 55 histologically normal samples, 48 (87%) were Normal-like, 6 (11%) were Luminal A, and 1 (2%) was Basal-like. Jackknife distances revealed that the sample with Basal-like subtype was a statistical outlier, suggesting that this normal sample may have had occult contamination with malignant cells that were not identified by pathologist review. The clinical relevance of normal tissue with invasive-like signatures is not established, but it is clear that some genes that are expressed in tumors are also variable in normal tissue.

We next defined a prototypical normal signature that could be used in the absence of a paired normal genomic profile to study the robustness of PAM50 genomic prediction for a given sample. To be used as a prototypical signature, expression must represent the majority of normal samples, thus we selected the 48 samples (representing approximately 90% of samples) that were classified as Normal-like; the prototypical normal was defined as the median gene expression across these Normal-like samples. The observation that some histologically normal tumors may be classified as invasive tumors using genomic predictors has been reported previously [11], so we also considered a prototypical normal based on the median of all 55 samples; at the 30% correction rate, the PAM50 subtype and ROR-S was identical for all samples in the NKI295. The correlation between the two prototypical normal profiles was 0.999. Thus, because the meaning of an invasive-like signature in adjacent non-neoplastic tissue is still poorly understood, we used a prototypical signature based on the 48 normal-like samples to perform adjustments. Using a value of *a *that represents the percentage of malignant cells in the tumor sample, we can rearrange equation 1, and subtract the effects of normal tissue (1-*a*) to obtain a corrected tumor signature:(2)

For the UNC24 dataset, *a *was set to equal the percentage of malignant nuclei in the sample. For the public datasets, we applied a single value of *a *for all tumors in a given dataset, with *a *chosen to maximize negative predictive value, while also ensuring that all ROR-S categories had sufficient sample size to provide stable survival analyses. Analyses of the UNC24 dataset were performed in an attempt to identify gene sets that could be used to predict percent normal in independent data and we evaluated a published percent normal signature [23], but neither had predictive accuracy in independent test sets, suggesting that while gene sets can be identified that distinguish normal from tumor, identification of gene sets that quantitatively estimate percentage normal is a more challenging problem. In the absence of a genomic method for estimating percent normal, we used pathologist assessment where available, or selected a uniform value of *a *across each dataset/population. With the latter method there is potential to 'over-adjust' or 'under-adjust' individual tumors, however the number of tumors that are adjusted 'more than necessary' will be approximately equal to those that are adjusted 'less than necessary'. Thus, despite the lack of individual-level tumor purity data in most public dataset, the overall population effects and survival estimates are well approximated and the population-level results illustrate the vulnerability of tumor prognostics to normal contamination. Furthermore, the estimated *a *values selected for each dataset are correlated with the reported quality control standards (see Results) for each dataset, suggesting validity of this approach.

### Survival analysis

Among the 295 patients with primary breast carcinoma in the NKI dataset, 151 were lymph node-negative and 36% of these node-negative individuals developed distant metastasis. For the Naderi et al.[13] and Wang et al.[12] datasets, 86 and 286 patients had lymph node-negative disease, respectively, from which 27.9% and 37.4% developed distant metastasis. We used PAM50 to classify all node-negative patients as low, medium or high risk of relapse (ROR-S) and compared these classifications to their metastasis event occurrences. For the NKI dataset 50 of 50 genes in the PAM50 were on the array. Likewise for Naderi et al.[13] and for Wang et al.[12], 48 and 44 of the PAM50 genes were available. We used the Survival package in R to perform univariate survival analyses (Kaplan-Meier Analyses).

### Sensitivity and negative predictive value

We used the sensitivity and negative predictive value (NPV) to evaluate the performance of PAM50 as a binary classifier of risk of relapse. We defined sensitivity as the probability of receiving a high or medium risk of relapse score (ROR-S), given relapse occurred by 5 years, by 10 years, or by the end of follow up. Negative predictive value was defined as probability of remaining disease free at a particular time point or at the end of follow up (overall) given a low risk score. The focus of the study was on sensitivity and NPV because these tests are used to guide decisions about chemotherapy and sensitivity (to detect those who will relapse) and negative predictive value (to identify patients who will not relapse) are the highest priority [33].

## Competing interests

The primary and corresponding author (FE and MT) have no financial relationship with any of the assays described in this paper. Likewise ZH, YL, MG, KA have no financial relationship with any of the assays described. MT and JP have an ongoing collaborative relationship with Charles Perou, corresponding author of several manuscripts on the PAM50 assay and author of a patent for a commercial version of this assay. JP provided bioinformatics consultation and is a coauthor on a patent of the commercial version of the PAM50, however, this manuscript does not assess the commercial version of the PAM50 or any of the other assays discussed.

## Authors' contributions

FE and MAT were responsible for the study design, data collection and analysis, interpretation of the results, and writing and final approval of the report. ZH, YL, MLG, and KDA were responsible for the provision of data, and writing and final approval of the report. JP provided bioinformatics consultation on application of the published version of the PAM50 and approved the final report. All authors read and approved the final manuscript.

## Pre-publication history

The pre-publication history for this paper can be accessed here:

http://www.biomedcentral.com/1755-8794/4/54/prepub

## Supplementary Material

Additional file 1 **Genes box plots for 48 tumor-adjacent samples with normal-like subtype**. Genes box plots for 48 tumor-adjacent samples with normal-like subtype. Genes are grouped according to their use in identifying breast cancer subtypes (i.e. 10 genes used to calculate Luminal A score together). Click here for file

Additional file 2 **PAM50 classification for the 55 patients assuming up to 50% contamination in Equation 1**. In A, we used the paired adjacent normal sample to perform the linear combinations and in B, the prototypical PAM50 normal signature was used in linear combinations. The PAM50 predictor was applied to the calculated gene expression under equation 1 in both scenarios. The agreement between A and B suggest that a prototypical normal signature can be used study the sensitivity of the PAM50 to normal contamination. Click here for file

Additional file 3 **Effect of correction for percentage normal in samples with known % normal**. Five of 24 samples had original subtype classifications that differed from those after correction for percentage normal determined with nuclei counts. Click here for file

Additional file 4 **Supplementary Data 1 showing sensitivity and negative predictive value at 10 years and to end of follow up**. Sensitivity and negative predictive values are given for 10-year and relapse-free survival at the end of follow up for NKI, Naderi, and Wang datasets. Click here for file

Additional file 5 **Overall survival plots for PAM50 ROR-S score, adjusted and unadjusted**. Overall survival plots for PAM50 ROR-S score given (A) Unadjusted NKI, (B) 30% normal-adjusted NKI and (C) Unadjusted Naderi et al., (D) 20% correction rate in Naderi et al. Corrections to the expression assuming a given percentage of tumor were calculated using equation 2. In each figure, the green line is low ROR-S, the light blue line is medium ROR-S and the red line is high ROR-S. Click here for file

Additional file 6 **Use of cell lines to test assumption of linear and symmetrical influence of epithelium and stroma on transcript abundance**. Expression data from pure populations of cancer cells grown in monoculture were linearly combined with expression data from pure populations of breast fibroblasts; expected expression values for ratios of epithelium to stroma equal to 3:1, 2:1 or 1:1 were computed. These computed values were compared to observed gene expression for cocultured cells where the actual percentage of epithelial content was 75%, 66% purity, or 50%, respectively. All data for these analyses are available in the Gene Expression Omnibus, GSE22384) and described in Camp et al. [32]. Supplemental Table 2 shows that the correlations between observed and computed were high (0.80-0.92) and that the slopes were very close to 1. Click here for file

Additional file 7 **Survival plots, unadjusted for normal contamination**. Survivals plots for (A) PAM50, (B) Prognosis method and (C) 21-gene assay on NKI node-negative patients, unadjusted for normal contamination. Consistent with previous findings, all three signatures are significantly associated with survival. Click here for file
