# Supplementary figures and images for: Systematic Bias in Genomic Classification Due to Contaminating Non-neoplastic Tissue in Breast Tumor Samples
Source: BMC Med Genomics. 2011 Jun 30;4:54. doi: 10.1186/1755-8794-4-54 (PMC3151208; doi:10.1186/1755-8794-4-54)

Figure S1.

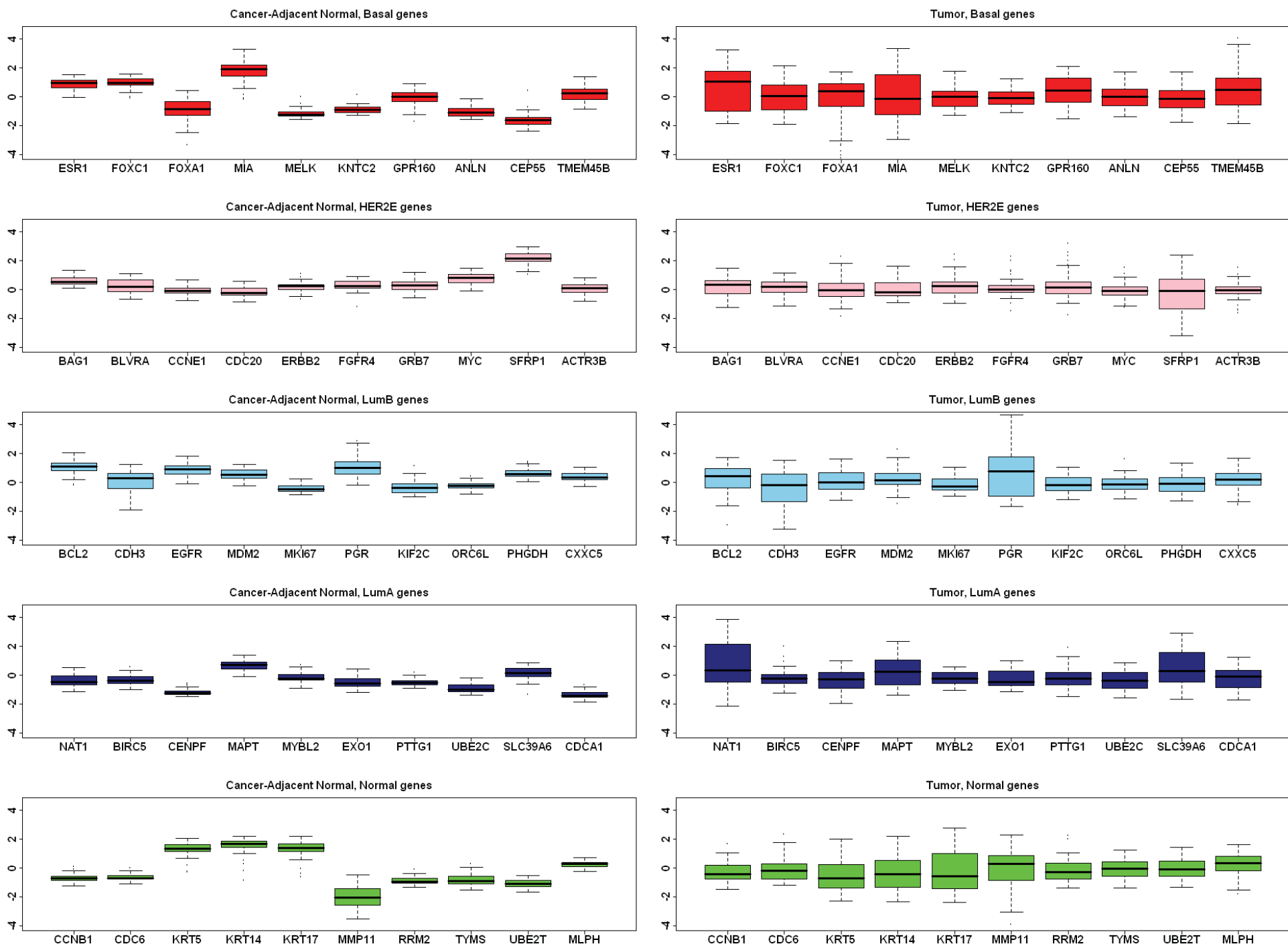

Supplement: Additional file 1 — Genes box plots for 48 tumor-adjacent samples with normal-like subtype. Genes box plots for 48 tumor-adjacent samples with normal-like subtype. Genes are grouped according to their use in identifying breast cancer subtypes (i.e. 10 genes used to calculate Luminal A score together). [file 1755-8794-4-54-S1.PDF]

Figure S2.

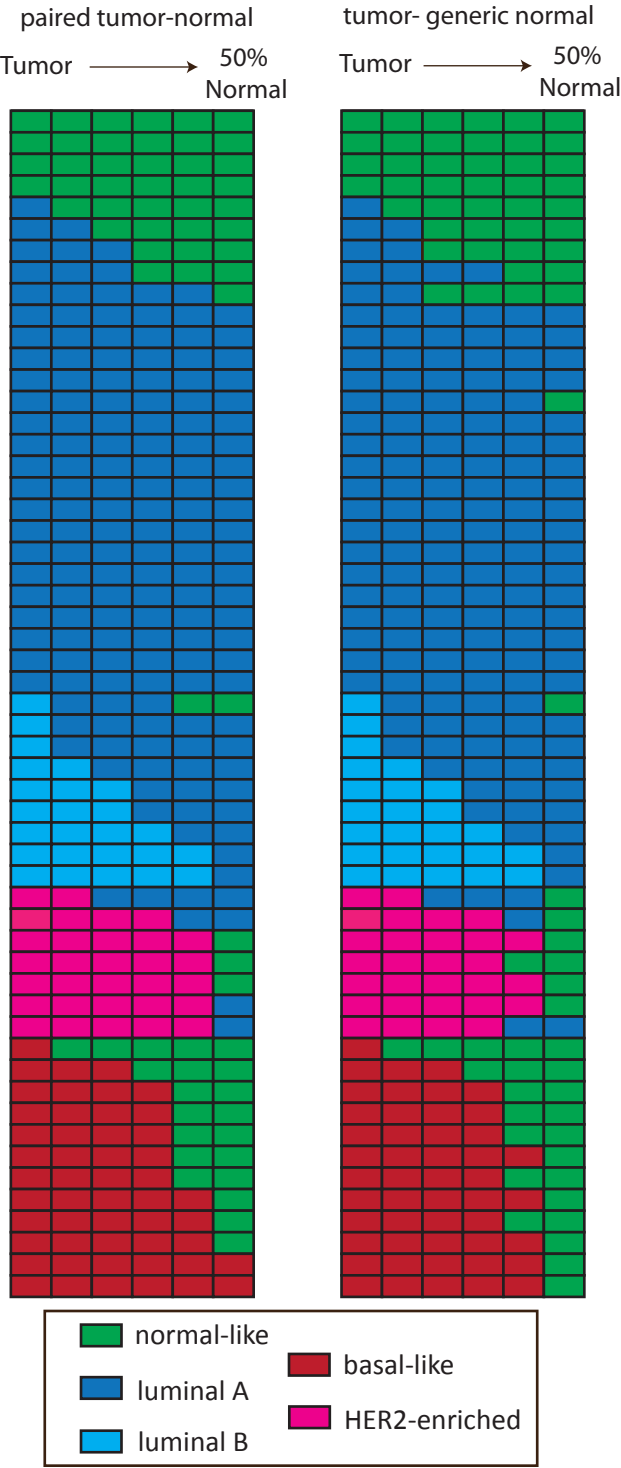

Supplement: Additional file 2 — PAM50 classification for the 55 patients assuming up to 50% contamination in Equation 1. In A, we used the paired adjacent normal sample to perform the linear combinations and in B, the prototypical PAM50 normal signature was used in linear combinations. The PAM50 predictor was applied to the calculated gene expression under equation 1 in both scenarios. The agreement between A and B suggest that a prototypical normal signature can be used study the sensitivity of the PAM50 to normal contamination. [file 1755-8794-4-54-S2.PDF]
