# Supplementary material for: Systematic Bias in Genomic Classification Due to Contaminating Non-neoplastic Tissue in Breast Tumor Samples
Source: BMC Med Genomics. 2011 Jun 30;4:54. doi: 10.1186/1755-8794-4-54 (PMC3151208; doi:10.1186/1755-8794-4-54)
Supplement: Additional file 3 — Effect of correction for percentage normal in samples with known % normal. Five of 24 samples had original subtype classifications that differed from those after correction for percentage normal determined with nuclei counts. [file 1755-8794-4-54-S3.DOCX]

Table S1. PAM50 Tumor Subtype on UNC 24 Specimens with Known Tumor Percentage, Unadjusted and Adjusted

|  | Tumor percentage | Unadjusted Subtype | Adjusted Subtype* | Unadjusted ROR-S | Adjusted  ROR-S |
| --- | --- | --- | --- | --- | --- |
| RAM97-1045B | 10 | Normal-like | Luminal B* | Low | High* |
| RAM02-7055B | 20 | Basal-like | Basal-like | High | High |
| RAM97-0137B | 20 | Basal-like | Basal-like | High | High |
| RAM95-0017B | 25 | Luminal B | Luminal B | Med | High* |
| RAM00-0587B | 30 | Luminal A | Luminal B* | Low | Med* |
| RAM95-0152B | 30 | HER2E† | HER2E† | Med | High* |
| RAM00-0572B | 30 | Basal-like | Basal-like | High | High |
| RAM02-7050B | 40 | Luminal A | Luminal A | Low | Low |
| RAM99-0348B | 50 | Luminal A | Luminal A | Low | Med* |
| RAM94-1096B | 50 | Luminal B | HER2E†* | High | High |
| RAM00-0365B | 60 | Luminal B | Luminal B | High | High |
| RAM98-0161B | 65 | Luminal B | Luminal B | Med | High* |
| RAM01-0349B | 70 | Luminal A | Luminal B* | Med | Med |
| RAM02-7035B | 70 | Basal-like | Basal-like | High | High |
| RAM95-0184B | 75 | Luminal A | Luminal B* | Low | Med* |
| RAM99-0622B | 75 | Luminal B | Luminal B | High | High |
| RAM03-7032B | 75 | Basal-like | Basal-like | High | High |
| RAM00-0344B | 80 | Luminal B | Luminal B | Med | High* |
| RAM01-0123B | 85 | HER2E† | HER2E† | High | High |
| RAM00-0504B | 90 | Luminal A | Luminal A | Low | Low |
| RAM03-7268B | 90 | Luminal B | Luminal B | Med | Med |
| RAM99-0207B | 90 | Luminal B | Luminal B | Med | Med |
| RAM00-0284B | 90 | HER2E† | HER2E† | High | High |
| RAM01-0246B | 100 | Luminal B | Luminal B | Med | Med |

*Indicates subtype changed relative to unadjusted data.

†HER2E refers to the HER2-enriched subtype as identified using microarray data.
