# Supplementary material for: Systematic Bias in Genomic Classification Due to Contaminating Non-neoplastic Tissue in Breast Tumor Samples
Source: BMC Med Genomics. 2011 Jun 30;4:54. doi: 10.1186/1755-8794-4-54 (PMC3151208; doi:10.1186/1755-8794-4-54)

Figure S3.

A. NKI, unadjusted ROR-S

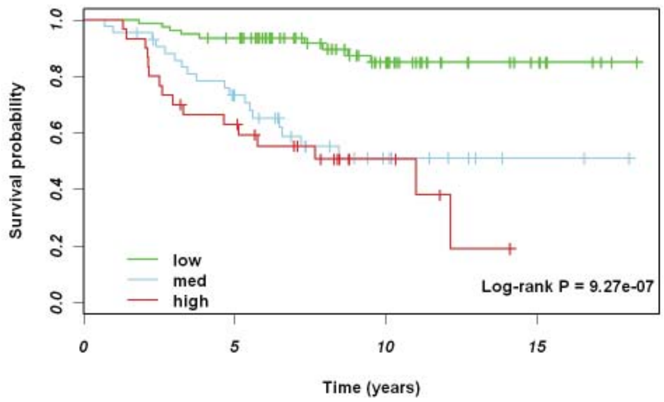

B. NKI, 30% normal-adjusted ROR-S

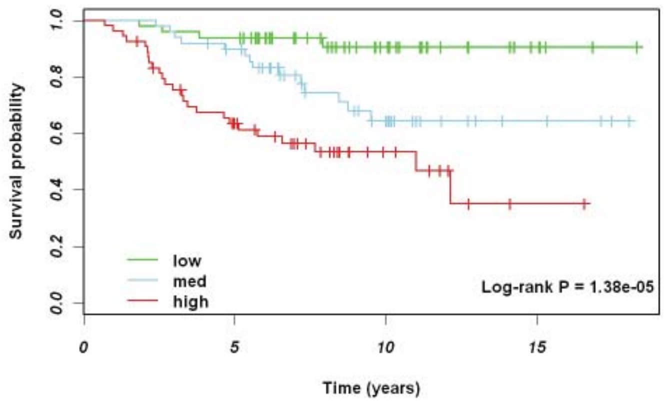

C. Naderi et al., unadjusted ROR-S

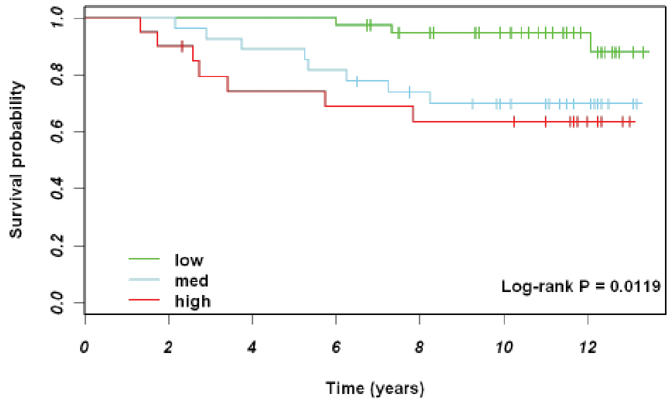

D. Naderi et al., 20% normal-adjusted ROR-S

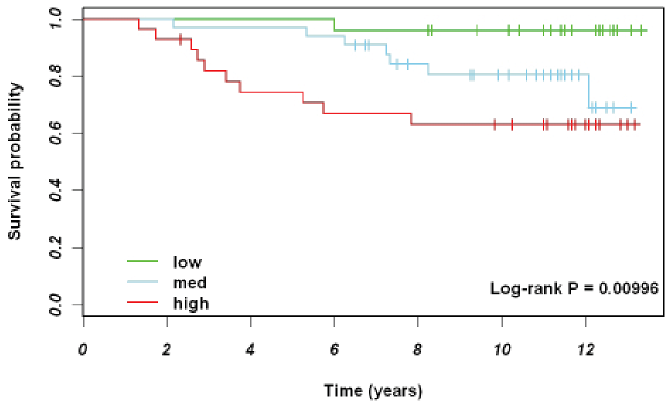

Supplement: Additional file 5 — Overall survival plots for PAM50 ROR-S score, adjusted and unadjusted. Overall survival plots for PAM50 ROR-S score given (A) Unadjusted NKI, (B) 30% normal-adjusted NKI and (C) Unadjusted Naderi et al., (D) 20% correction rate in Naderi et al. Corrections to the expression assuming a given percentage of tumor were calculated using equation 2. In each figure, the green line is low ROR-S, the light blue line is medium ROR-S and the red line is high ROR-S. [file 1755-8794-4-54-S5.PDF]
