# Supplementary material for: Systematic Bias in Genomic Classification Due to Contaminating Non-neoplastic Tissue in Breast Tumor Samples
Source: BMC Med Genomics. 2011 Jun 30;4:54. doi: 10.1186/1755-8794-4-54 (PMC3151208; doi:10.1186/1755-8794-4-54)
Supplement: Additional file 6 — Use of cell lines to test assumption of linear and symmetrical influence of epithelium and stroma on transcript abundance. Expression data from pure populations of cancer cells grown in monoculture were linearly combined with expression data from pure populations of breast fibroblasts; expected expression values for ratios of epithelium to stroma equal to 3:1, 2:1 or 1:1 were computed. These computed values were compared to observed gene expression for cocultured cells where the actual percentage of epithelial content was 75%, 66% purity, or 50%, respectively. All data for these analyses are available in the Gene Expression Omnibus, GSE22384) and described in Camp et al. [32]. Supplemental Table 2 shows that the correlations between observed and computed were high (0.80-0.92) and that the slopes were very close to 1. [file 1755-8794-4-54-S6.DOCX]

Table S2. Slope of Univariate Linear Regression Near 1 and High Pearson Correlations Demonstrate Good Agreement between Observed and Computed Gene Expression

|  | Slope (95% confidence interval) | Pearson Correlation |
| --- | --- | --- |
| 75% epithelium, replicate 1 | 0.82 (0.70 – 0.93) | 0.89 |
| 75% epithelium, replicate 2 | 1.03 (0.91 – 1.15) | 0.92 |
| 66% epithelium, replicate 1 | 1.10 (0.96 – 1.24) | 0.91 |
| 66% epithelium, replicate 2 | 1.22 (1.04 – 1.41) | 0.88 |
| 50% epithelium, replicate 1 | 1.01 (0.76 – 1.25) | 0.75 |
| 50% epithelium, replicate 2 | 1.37 (1.08 – 1.65) | 0.80 |
