# Supplementary material for: Systematic Bias in Genomic Classification Due to Contaminating Non-neoplastic Tissue in Breast Tumor Samples
Source: BMC Med Genomics. 2011 Jun 30;4:54. doi: 10.1186/1755-8794-4-54 (PMC3151208; doi:10.1186/1755-8794-4-54)

Figure S4.

A - PAM50 on Node Negative NKI patients

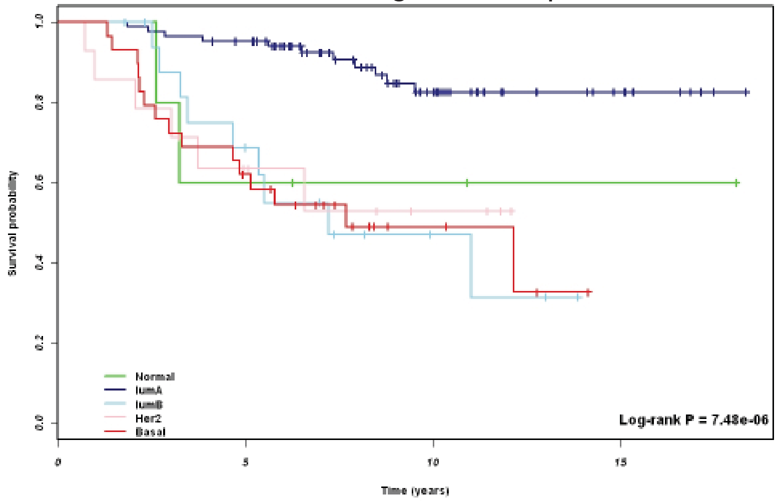

B - Prognosis Score on Node Negative NKI patients

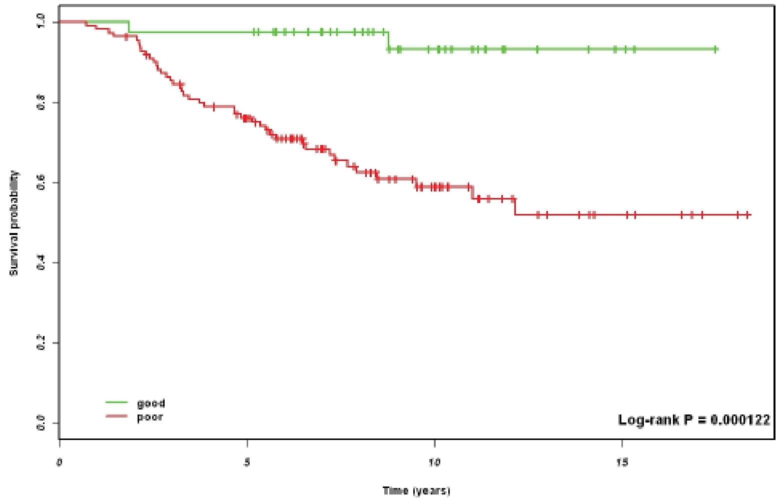

C - Recurrence Score on Node Negative NKI patients

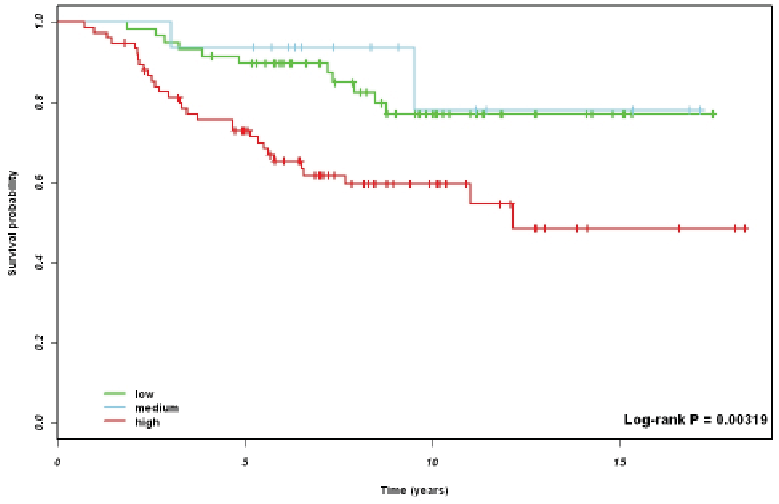

Supplement: Additional file 7 — Survival plots, unadjusted for normal contamination. Survivals plots for (A) PAM50, (B) Prognosis method and (C) 21-gene assay on NKI node-negative patients, unadjusted for normal contamination. Consistent with previous findings, all three signatures are significantly associated with survival. [file 1755-8794-4-54-S7.PDF]
